# A Comprehensive Review of the Genomics of Multiple Myeloma: Evolutionary Trajectories, Gene Expression Profiling, and Emerging Therapeutics

**DOI:** 10.3390/cells10081961

**Published:** 2021-08-02

**Authors:** Hassan Awada, Bicky Thapa, Hussein Awada, Jing Dong, Carmelo Gurnari, Parameswaran Hari, Binod Dhakal

**Affiliations:** 1 Department of Translational Hematology and Oncology Research, Taussig Cancer Institute, Cleveland Clinic, Cleveland, OH 44106, USA; awadah@ccf.org (H.A.); awadah3@ccf.org (H.A.); carmelogurnari31@gmail.com (C.G.); 2 Division of Hematology and Oncology, Medical College of Wisconsin, Milwaukee, WI 53226, USA; bithapa@mcw.edu (B.T.); jidong@mcw.edu (J.D.); phari@mcw.edu (P.H.)

**Keywords:** multiple myeloma, plasma cell dyscrasia, genomics, risk stratification, prognosis

## Abstract

Multiple myeloma (MM) is a blood cancer characterized by the accumulation of malignant monoclonal plasma cells in the bone marrow. It develops through a series of premalignant plasma cell dyscrasia stages, most notable of which is the Monoclonal Gammopathy of Undetermined Significance (MGUS). Significant advances have been achieved in uncovering the genomic aberrancies underlying the pathogenesis of MGUS-MM. In this review, we discuss in-depth the genomic evolution of MM and focus on the prognostic implications of the accompanied molecular and cytogenetic aberrations. We also dive into the latest investigatory techniques used for the diagnoses and risk stratification of MM patients.

## 1. Introduction

Multiple myeloma (MM), is a malignant clonal proliferation of plasma cells compromising 1.8% of all new cancer cases in the U.S. based on the Surveillance, Epidemiology, and End Results Program (SEER) cancer database [1]. Data from randomized clinical trials revealed an overall five-year survival rate of about 54% and a median overall survival of approximately six years [2]. Most recently, a study on long-term outcomes in MM after autologous stem cell transplantation revealed better overall survival in patients treated in 2014 or after as compared to 1997 or before [3]. This significant difference in outcomes derives from the substantial progress made in the understanding of disease pathobiology and the introduction of novel therapeutics [4,5]; yet prognosis remains poor, especially in genetically defined high-risk subgroups [6]. MM is a heterogeneous disease characterized by the acquisition of complex genetic changes during disease evolution from the premalignant condition monoclonal gammopathy of undetermined significance (MGUS) [7] and smoldering MM (SMM) [8]. Seminal studies provided crucial information about the complex evolutionary process in MM patients and changes in genomics of clonal architecture as the disease progresses [9,10,11]. In clinical practice, conventional karyotyping and interphase fluorescence in situ hybridization (FISH) from bone marrow samples are utilized to classify genomic risk and identify patients with high-risk abnormalities [8]. Recently, extensive studies of MM genomics have led to an improved understanding of the molecular biology of MM. This resulted in new molecular classifications of MM subtypes, that have been proposed using gene expression profiling, and the identification of genes involved in the disease process [12,13]. Here, we summarize the current knowledge on the complex genomic landscape and pathophysiological mechanisms of MM.

## 2. Genetic and Cytogenetic Abnormalities: A Long Way from MGUS to MM Progression

**Figure 1:**
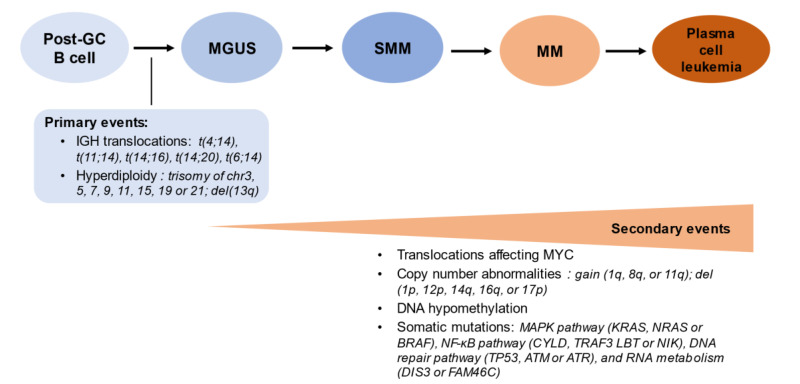
The sequence of genomic events in the molecular pathogenesis of multiple myeloma.

The genetic complexity of MM underlies a progressive, multistep process through which the continuous accumulation of genetic aberrancies drives monoclonal plasma cells towards malignancy [14]. These aberrancies are generally classified as either primary or secondary (Figure 1). Primary aberrancies initiate the process of plasma cell immortalization and its consequent commitment to the MM disease pathway [15]. Whether its progress eventually leads to MM or halts/remains at an earlier phase is dependent on the secondary aberrancies which further modulate disease progression [15]. It is now known that MM, in almost all patients, evolves from a premalignant precursor stage, MGUS, which is characterized by monoclonal plasma cells of limited malignant potential. Hence, to fully understand the genetic foundations of the pathophysiology of MM, we must also consider the pathogenesis of MGUS, its direct precursor. Several studies investigated the genomic changes occurring at the stage of MGUS [16,17]. 

Plasma cells are terminally differentiated B cells, which in physiologic conditions are incapable of undergoing cell division. The acquisition of genomic aberrations (cytogenetics primary events, left panel) may underpin the transformation from polyclonal to monoclonal plasma cells (stage of monoclonal gammopathy of undetermined significance, MGUS). Additional molecular events underlie the progression to smoldering multiple myeloma (SMM), multiple myeloma (MM) with clinical disease manifestations, and at the extreme pole, plasma cell leukemia when >2 × 10^9^ plasma cells/L are present in the peripheral blood (or >20% of nucleated blood cells are constituted by plasma cells) [18].

In fact, terminally differentiated plasma cells do not undergo cell division, however, epigenetic deregulation of gene expression has been proposed as a root cause of malignant transformation in MM [19]. Genome-wide association studies (GWAS) by Broderick and his team identified germline variants at 3p22.1, 7p15.3, and 2p23.3 as risk factors for developing MGUS [15,20]. Their associated gene pairs (*DNMT3A* and *DTNB, *ULK4** and *TRAK1, *DNAH11** and *CDCA7L,* respectively) were found to be implicated in the dysregulation of the transcription factor encoding the MYC proto-oncogene [21]. Beksac et al. investigated the association of different human leukocyte antigen (HLA) polymorphisms with the risk of developing MM [22]. While the authors confirmed DRB5*01, C*07:02 g and B*07:02 g as potential risk-alleles for MM, they also illustrated their high correlation by linkage disequilibrium in Whites. Moreover, the cross-population analysis suggested that C*07 represents the only independent risk-allele for MM while the other alleles occurring in the same haplotype rather than contributing to MM [22]. This analysis also demonstrated the protective associations of C*05:01 g and B*44:02 g when occurring on the same haplotype in Whites, and the predisposing associations of C*12:03 g~B*38:01 haplotype in Whites while B*58:01 g in Asians [22]. Nonetheless, somatic mutations and cytogenetic abnormalities are bigger contributors along the MGUS-MM clinical spectrum.

### 2.1. Primary Events Driving MGUS Progression to MM

Cytogenetic abnormalities split MM cases into two broad divisions: the hyperdiploid and nonhyperdiploid subtypes. These two subtypes differ in the type of the primary cytogenetic aberrancy that drives the plasma cells towards the MGUS-MM pathway. Hyperdiploidy in MGUS-MM involves the acquisition of one or more odd-numbered chromosomes in a clonal cell population, including chromosomes 3, 5, 7, 9, 11, 15, 17, and 19 [23,24,25,26]. The extra chromosomes carry genes whose overexpression may promote dysregulated replication and growth, transforming normal cells into an MGUS-MM clone. 

On the contrary, the nonhyperdiploid subtype is mainly characterized by the translocation of the immunoglobulin heavy chain (IgH) locus on 14q32, which juxtaposes an oncogene on the affected recipient chromosome [24,27]. Consequently, the expression of the juxtaposed oncogene would now be under the influence of the active upstream IgH promoter [27]. The most commonly affected genes are that of cyclin D1 (11q13), cyclin D3 (6p21), FGFR3 and MMSET (4p16), c-maf (16q23), as well as mafB (22q11) [28,29]. Thus, a variety of transcription factors, growth factor receptors, and other cell cycle mediators may become overexpressed and dysregulate the plasma cell cycle, which may lead to cellular proliferation. 

Although the triggers of these primary cytogenetic aberrancies remain unclear, some reports have suggested it is due to an abnormal response of plasma cells to antigenic stimulation [30,31]. Chronic antigenic stimulation predisposes to IgH class-switching recombination errors that could possibly lead to these translocations [15]. While the links remain to be fully clarified, several studies have demonstrated that the overexpression of Toll-like receptors (TLRs) and the interleukin 6 (IL-6) receptor on the surface of plasma cells promotes their proliferation, survival, and resistance to apoptosis [31,32,33]. IL-6, in particular, has been implicated in the expression of the anti-apoptotic proteins Mcl-1 and Bcl-Xl [34]. However, the sequence of events by which chronic stimulation of these receptors induces the cytogenetic aberrancies needs to be deciphered in order to fully elucidate the pathogenesis of MM. 

### 2.2. Secondary Events Driving MGUS Progression to MM 

Once the MGUS clone is established, secondary aberrancies dictate its transformation into MM or not [35]. These secondary events are generally thought to follow the random “second hit” model. In particular, this model provides the best explanation for the consistent—rather than the cumulative—annual rate (1%) of MGUS transformation into MM [7]. However, this long-standing belief has been contested recently by a study demonstrating that the individual risk of developing MM may increase over time, but in patients with MGUS diagnosed in the setting of immune-related disorders, the risk of progression could be lower [36,37]. Physicians usually determine the risk of transformation shortly after diagnosis with MGUS, but repeated reassessments may allow researchers to recognize that some of the “high risk” cases were “low risk” in previous tests. This study further emphasized the lack of certainty of models assessing MGUS progression into MM. 

The majority of secondary events are also of a genetic/cytogenetic origin, which include translocations, deletions, mutations, and others. Secondary translocations are class-switching independent as opposed to primary translocations [38]. They mainly affect *MYC* (8q24), whose overexpression is associated with late disease progression and poor prognosis [29,39]. It is uncommonly seen in MGUS, yet witnessed in 15% of MM and 50% of advanced disease cases [15,40]. MYC is frequently dysregulated by t(8;14) involving the IgH locus at 14q32, but around 40% of the MYC translocations do not involve an immunoglobulin (Ig) locus [38]. Moreover, the pattern of its occurrence, with or without Ig loci involvement, is similar across both hyper and nonhyperdiploid MM, and its expression is alike regardless of whether an Ig or non-Ig enhancer is involved [39,41]. Interestingly, the presence of MYC rearrangements has been recently recognized as an independent adverse prognostic factor in newly diagnosed patients with MM [42].

The deletion of the tumor suppressor gene *TP53* at 17p13 is present in 10% of newly diagnosed MM cases [43]. Its protein product, p53, functions as a transcriptional regulator that surveils for DNA damage, prompting cell cycle arrest and apoptosis if DNA repair fails. In addition, monoallelic 17p13 deletion increases the risk of mutation of the remaining TP53 allele by 37%, which explains why hemizygous patients are at a greater risk of rapid disease progression and may end up with plasma cell leukemia or central nervous system MM [44,45,46]. The prognostic effect of the 17p deletion cancer clonal fraction (CCF) (fraction of cancer cells carrying the deletion) has also been of research interest, as Thakurta and his team demonstrated in their work that a threshold CCF of 0.55 of this deletion is indicative of poor prognosis and shorter survival [47]. In contrast, patients with del17p CCF ≤0.55 have comparable clinical outcomes to those with wild-type TP53, while double-hit patients (biallelic deletions) have worse outcomes compared to monoallelic deletions regardless of the CCF category [47].

*Ras* mutations also promote the progression of MGUS into MM, and these oncogenes are mutated in up to 40% of newly diagnosed MM cases [48]. Indeed, *KRAS* mutations are significantly associated with TP53 mutation and cyclin D1 t(11;14) as opposed to other primary IgH translocations, while *NRAS* mutations significantly decrease disease sensitivity to bortezomib therapy [49,50]. Both RAS mutations are associated with poor prognosis, aggressive disease phenotype and lower survival rates [50].

The NF-kB pathway has also been implicated in the pathogenesis of MM, as its expression has been found to be constitutively active in at least 50% of MM cases [51]. Its role is evident in the pathogenesis of both MGUS and MM, and hence can be thought of as an aberrancy along the entire MGUS-MM pathway [52]. NF-Kb promotes the survival of plasma cells, and the gain of function mutations along its signaling pathways result in the malignant accumulation of these cells beyond physiological control [53]. 

Several other factors also increase the burden of the progression of MGUS into MM. Loss of function of cyclin-dependent kinase inhibitors (CdkI) through hypermethylation or deletions leads to the loss of negative regulation over the cell cycle [54]. Along with the aberrant expression of cyclins D1-3 from IgH translocation, these alterations lead to cell-cycle dysregulation, increased proliferation rate, and are associated with poor survival [54]. Finally, the overexpression of IL-6 and the anti-apoptotic protein Bcl-2, mutations in the Fas-FasL pathway, and hypermethylation of the apoptotic death-associated protein (DAP) kinase have been implicated in prolonged survival and the malignant accumulation of plasma cells [55,56,57,58].

## 3. Fluorescent In-Situ Hybridization (FISH)

FISH is the current gold standard for identifying the genomic abnormalities in MM, which may predict the aggressiveness of the disease [59]. Prognostic abnormalities that can be detected by FISH include the trisomies of odd-numbered chromosomes, IgH translocations, 17p13 and 1p32 deletions, and 1q21 amplification. 

FISH offers a far greater degree of sensitivity and specificity compared to traditional methods like G-banding karyotyping, which can detect only 20–30% of the cytogenetic abnormalities in aspirated bone marrow plasma cells and is hindered by the need of having the plasma cells in metaphase [59]. FISH, in contrast, requires the cell to be in interphase, the phase of the cell cycle in which the majority of cells are at in a given point of time [59]. FISH detection rate of cytogenetic abnormalities in plasma cell dyscrasia can further be enhanced with plasma cell enrichment, by increasing the number of the plasma cells collected in the bone marrow aspirate undergoing analysis [60,61,62].

FISH is also one of the main components of the Revised International Staging System (R-ISS) for MM [63] (Table 1). R-ISS is a simple and powerful prognostic staging system that is universally applicable since it takes into consideration clinical and laboratoristical information and FISH markers that are largely available in most treatment centers as opposed to the Mayo clinic risk stratification for MM (mSMART) [64]. It combines the elements of the original ISS of MM (serum beta-2 microglobulin (B2M) and albumin), chromosomal abnormalities detected by FISH, and serum LDH levels in order to create a tool that can effectively determine the relative risk of survival of newly diagnosed MM patients [63]. Depending on the measurements of these elements, patients are categorized as R-ISS I, II, or III, with R-ISS III having the worst overall survival (OS) and progression-free survival rates [63]. This system considers anyone with serum B2M ≥5.5 mg/L and elevated LDH to be of stage III, with or without del 17p13, t(4;14) or t(14;16) [63]. On the contrary, R-ISS I patients lack any of these cytogenetic anomalies and have serum B2M < 3.5 mg/L, serum albumin ≥3.5 g/dL and normal LDH levels [50]. Patients who do not meet the R-ISS I or III criteria are classified as stage II [50]. In the original study by Palumbo et al., median OS was not reached, 83 months and 43 months for R-ISS I, II, and III, respectively [50], while the progression free survival was 66 months, 42 months, and 29 months, respectively [50]. Furthermore, the R-ISS system can predict OS independent of age and therapy, and its reliability has been validated in the real-world setting [50].

## 4. Current Data on Co-Existing Abnormalities

The cytogenetic abnormalities underlying MM tend to occur in various combinations, each with distinct prognostic implications. Hence, to overcome the intrinsic limitations of R-ISS, which only accounted for three high-risk FISH abnormalities, Perrot et al. developed and validated a cytogenetic prognostic index (PI) that is based on the occurrence of cytogenetic abnormalities in a large cohort of 1635 MM patients enrolled in four trials [65]. They used FISH and SNP to investigate the effect of del(17p), t(4;14), del(1p32), 1q21 gain and trisomies 3, 5 and 21 on survival outcomes. Six of these abnormalities were statistically significant (all except trisomy 3) based on a multivariate cox proportional hazard regression model, from which a PI formula was derived: 0.4 × t(4;14) + 1.2 × del(17p) − 0.3 × trisomy 5 + 0.3 × trisomy 21 + 0.5 × 1q gain + 0.8 × del(1p32). Accordingly, low, intermediate and high-risk groups are identified by a PI score of ≤0, 0–1, and >1, respectively with an estimated 5-year survival of >75%, 50–75% and <50%. The most common association was reported between trisomies 5 and 21, followed by 1q21 gain with t(4;14) or del(1p32). Interestingly, the PI score had a higher C-index compared to the R-ISS, further demonstrating its discriminative and prognostic abilities. 

The concept of double hit and triple hit MM further represents another approach to risk-stratify newly diagnosed MM patients based on their number of high-risk abnormalities such as t(4;14), t(14;16), t(14;20), del(17p), p53 mutation, gain (1q) and del(1p) [66]. Double and triple hit MM are defined by having two and three or more of these high-risk genetic abnormalities, respectively. As expected, patients with double hit MM have been shown to have a worse prognosis than patients with only one high-risk genetic abnormality, while triple hit MM patients tend to have the poorest outcome of all [67,68]. Walker et al. defined double hit myeloma as the subgroup harboring either bi-allelic TP53 inactivation or amplification (≥4 copies) of CKS1B (1q21) on the background of clinical International Staging System III disease. 

## 5. Gene Expression Profiling

Despite its undoubtedly clinical utility, FISH is limited by the identification of only known cytogenetic abnormalities and the inability to decipher the molecular heterogeneity among patients. To this end, gene expression profiling (GEP) is a tool that helps us understand the biology of MM broadly by identifying genes involved in molecular pathogenesis and their clinical significance. Historically, GEP studies led to the identification of Cyclin D family deregulation in MM and MGUS [69,70,71]. In addition, several GEP studies have identified genes and pathways, which lead to the recognition of the molecular complexities involved in MM pathogenesis. Indeed, 11 different molecular subgroups of MM have been found based on transcriptomic studies [13]. Paralleling the differences in GEP, the different subgroups of MM also correlated with clinical outcomes. For instance, subclassifying MM patients according to the presence of IgH translocations and dysregulation of cyclin D genes (i.e., the translocation and cyclin D (TC) classification) allowed the identification of eight subgroups of MM (11q13, 6p21, 4p16, *maf*, D1, D1+D2, D2, and none) [69,70,71]. Another classification method from the University of Arkansas for Medical Sciences (UAMS) characterized seven different molecular subgroups of MM from 414 newly diagnosed MM based on activating translocations and hyperdiploidy [72]. These seven subgroups of myeloma are namely CD1 [(t(11;14)], CD2 [t(11;14) & t(11;16)], MS [t(4;14)], MF [t(14;16) & t(14;20)], Hyperdiploid cluster (HY), low bone disease (LB), and proliferation-associated genes (PR). The authors also identified myeloid gene expression signatures but were excluded from profiling analyses [72]. Most interestingly, CD1, CD2, LB, and HY subgroups were enriched in low-risk diseases with better overall survival outcomes while MS, MF, and PR subgroups were recognized as high-risk groups. Furthermore, the analyses of data from MM patients enrolled in the HOVON65/GMMG-HD4 trial were described in a European study that identified three additional subgroups of MM [73] including the nuclear factor kappa light chain-enhancer (NF-kB) subgroup, the Cancer testis antigen (CTA) subgroup characterized by high proliferation index, and the PRL3 subgroup characterized by up-regulation of protein tyrosine phosphatases *PRL-3* and *PTPRZ1*.

### Brief Summary of Genomic-Based Risk Stratification Studies

UAMS study: Initial study by the UAMS group identified a 70-gene high-risk signature from a training cohort of 351 newly diagnosed MM patients at times of diagnosis and relapse [74]. Investigators performed supervised clustering with 70 genes related to plasma cells from 14 MGUS patients, 22 healthy donors, 38 human MM cell lines, and 351 patients of the training cohort. Gene expression clustergram demonstrated high-risk groups with similar patterns as human MM cell lines, whereas low-risk MM groups exhibited patterns identical to MGUS and normal plasma cells. Further evaluation of the 70-gene risk model in relapse samples of 51 out of 351 of the training cohort revealed high-risk scores in 39 patients, which were associated with poor survival. Interestingly, 30% of the 70 high-risk genes were located in chromosome 1, shaping the high-risk profile of MM. Gene mapping studies revealed overexpressed genes at 1q21, 1q22, and 1q43-q44 in high-risk MM. Additionally, four other genes were identified in the 8q21-8q24 region in patients with high-risk features. Furthermore, the investigators identified a minimum of 17 genes out of the 70-gene model capable of distinguishing high-risk and low-risk MM. Of note, the 17-gene model predicted with 97.7% and 96.9% accuracy, the correct risk category assignment (high-risk vs. low-risk) in the training and the validation cohorts, respectively.

Skyline 92-HOVON study: Kuiper et al. identified 92-gene signatures (EMC-92) from newly diagnosed MM patients in the HOVON65/GMMG-HD4 trial that proved to be an independent prognostic factor for survival [75]. The authors only identified two overlapping genes, called *BIRCS5* & *LTBP1*, compared to UAMS-17/70 gene signatures. 

Intergroupe Francophone du Myelome identified 15 genes (IFM-15) from 250 newly diagnosed MM patients that were associated with poor prognosis [76]. The study found overexpression of genes involved in the cell cycle progression and its surveillance in high-risk MM patients. In IFM-15 model, only one gene (*FAM49A*) was common compared to EMC-92 model, albeit none with UAMS-70. 

Other models for prognostication include six gene expression signatures by Dickens et al. [77], the millennium signature [78], and GEP based proliferation index [79]. Despite the identification of various gene models for prognostication as described above, there is no consensus so far in routine clinical practice to incorporate GEP in MM management. Considering its costs, GEP is mostly utilized for research purposes than in routine clinical practice. In addition, while most of the previous studies utilized micro-array-based GEP, recently RNA-sequencing-based methods were found to be highly sensitive and specific, providing new avenues for MM patients prognostications [13]. 

## 6. DNA Sequencing and Data on Mutations 

As mentioned, given the remarkable clinical and biological heterogeneity of MM, FISH testing cannot capture the genomic complexity of the disease [80]. In addition, common primary events, such as hyperdiploidy and IgH translocations, are insufficient to drive overt MM [81]. Subsequently, secondary genetic events, such as translocations affecting *MYC*, copy number abnormalities, DNA hypomethylation and somatic mutations in oncogenic pathways (e.g., MAPK, NF-κB and DNA-repair), are required to drive tumor progression [8,82,83,84,85], often defining the true malignant potential but also serving as potential actionable therapeutic targets. 

**Figure 2:**
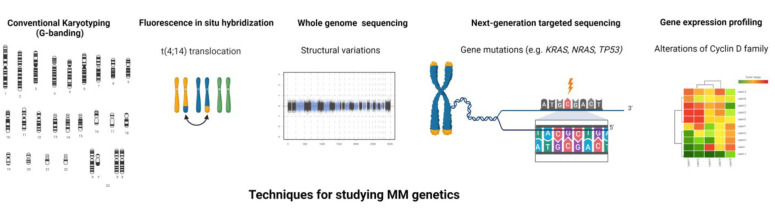
Techniques used for studying the genomics of multiple myeloma. The figure illustrates the various techniques used for the genomic dissection of multiple myeloma.

To this end, the use of new high throughput NGS techniques has markedly advanced our knowledge of MM biology (Figure 2). Since the first MM whole genome sequencing (WGS) study [81], an increasing number of recurrent mutations and structural variations (SVs) have been characterized in MM [86]. Although two-thirds of MM patients carry translocations in either IgH or MYC, a recent MM WGS study showed that there were more than 2000 SVs present in MM with IgH and MYC translations only accounted for 6.5% of all SVs [87], suggesting the importance of SVs as major drivers of MM development and progression. Among them, chromothripsis was the most frequent SVs, followed by chromoplexy [87]. Overall, an average of 1.6 mutations per Mb were observed in MM but no universal driver mutations were identified, with the most frequently mutated genes reported being *KRAS* and *NRAS* (in ~20% of patients each), followed by *FAM46C* and *DIS3* (~11% each), *TP53* (8%) and *BRAF* (6%) [8,82,83,84,85,87,88]. All other mutations (e.g., *TRAF3*, *LTB* and *ATM*) were observed in less than 5% of MM patients [8]. These mutations affect several signaling pathways with some patients carrying two or more mutations in genes pertaining to the same pathway (e.g., *KRAS*, *NRAS* and *BRAF* in the MAPK pathway) [83]. Beyond these well-defined myeloma genes, recent NGS-based studies have identified several other mutated genes, such as linker histones (*HIST1H1B*, *HIST1H1D*, *HIST1H1E*, and *HIST1H2BK*) [87], *FUBP1 (MYC* transcription regulator) and *MAX* (*MYC* DNA binding partner) [87], and non-coding mutations in the *cis*-regulatory elements (e.g., *HOXB3*, *PAX5* and *TPRG1*) [85]. Chronological reconstruction of genetic events showed that MM development follows preferred evolutionary trajectories [87]. While the majority of somatic mutations occur later in MM, some mutations, such as in activation-induced cytidine deaminase (AID) target genes [89], consistently occur in the early stages of tumor evolution. Driver events accumulated over time, but the resulted clinical course is unpredictable given the clonal evolution and heterogeneity of MM. Efforts have been made to leverage genomic information for MM precision medicine. For example, studies that combine mutations in *TP53*, *ATM* or *ATR*, *ZFH4* or *CCND1*, del(17p), t(4;14), amp(1q), and translocations involving MYC observed improved sensitivity for MM early detection and prognosis prediction, as compared to ISS [84]. The identification of these alterations also led to the development of targeted therapies to treat MM patients carrying specific driver mutations, such as Selumetinib (*KRAS*), Cobimetinib (*NRAS*), Palbociclib [del(1p), t(11;14) or t(6;14)], and Vemurafenib (*BRAF*) [90,91,92,93,94]. However, the genomic complexity of MM makes it challenging to find effective therapies and the “one size fits all” approach is inapplicable. More detailed assessment with genomic sequencing is needed for MM management, particularly because the majority of driver events can only be detected by WGS. Sequential samples (i.e., before and after progression or treatment) with both bone marrow and blood-based genetic profiling will be required in future studies to extend our knowledge of subclonal evolution and resistant clones. 

## 7. Concept of Clonal and Subclonal Evolution 

Clonal heterogeneity is a well-known phenomenon in MM contributing to the disease complexity. This puzzling characteristic is present both at the inter-patient and intra-patient levels, which adds to the intricacy in disease biology. In addition, the observed complexity in the clonal architecture of high-risk cytogenetic MM suggests a Darwinian-like somatic evolution rather than conventional linear evolution pattern [95,96,97,98]. Furthermore, the Darwinian model of tumor evolution explains the reason for the failure of therapeutic interventions [96]. Three landmark studies in 2012 provided insight into the complexity of the MM genomics landscape and its evolutionary process [9,10,11]. These studies demonstrated intraclonal heterogeneity early at the diagnosis and different stages of the disease after relapse. Egan et al. were the first to study the longitudinal evolution of myeloma to identify genomic changes during disease course by utilizing WGS data from longitudinal samples of a MM patient with t(4:14) [11]. The authors identified ten common single nucleotide variants (SNVs) that were shared at any point during the disease course. Interestingly, genomic variants were identified at alternating time points of the disease course suggesting the waxing and waning of different clones with treatment and disease status. The WGS study, in this case, demonstrated complex clonal dynamics with genomic heterogeneity at the different studied time points and clonal tiding during the disease course suggesting a Darwinian model of tumor evolution in MM. 

Later on, Keats et al. studied genomic and clonal dynamics in 28 MM patients at different time points during the disease course [9]. The authors described three distinct patterns of genomic evolution: genetically stable, linearly evolving clones, or shifting predominant clones. In about one-third of patients with standard-risk (low-risk hyperdiploid disease) cytogenetics, they observed stable genomes with few changes over time and favorable clinical outcomes. Another two-thirds was comprised of high-risk cytogenetics and was characterized by genomic instability with an increased propensity to change over time. Some of the cases in the high-risk group acquired new copy number alterations (CNAs), suggesting a traditional model of linear evolution. However, most of the high-risk MM cases were found to have multiple unique clones at initial diagnosis with changes in relative frequency over time. In particular, MM with high-risk cytogenetics such as t(4;14), t(14;16), t(14;20), and del(17p13) was associated with increased CNAs over time and patients with del(17p13) had significantly more CNAs at diagnosis as compared to other high-risk cytogenetics. A further longitudinal investigation of patients carrying del(17p13) revealed loss of *TP53* alleles in four out of five studied patients. Thus, biallelic inactivation over time potentially represents the molecular underpinning of the observed poor prognosis of del(17p13) cases. Like Egan et al., the authors also explored the tumor genomics at different time points in one patient with t(4:14), and they observed alternating clonal dominance [9,11]. Therapeutic interventions over the disease course were associated with clonal suppression and recurrence, which seemed to correlate with drug sensitivity and resistance. 

**Figure 3:**
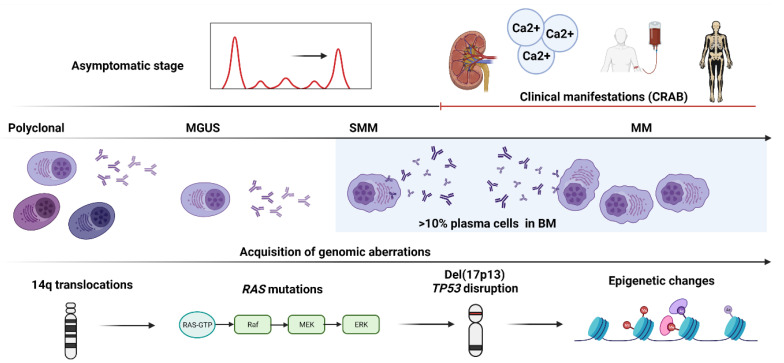
The step-wise acquisition of genomic aberrations in a patient with multiple myeloma. The figure shows a schematic representation of the stepwise acquisition of genetic aberrations in an exemplary patient with multiple myeloma (MM) from the preclinical/asymptomatic stage (monoclonal gammopathy of undetermined significance, MGUS), to smoldering MM (SMM) and later to the clinical/symptomatic stage characterized by typical manifestations of calcium elevation, renal dysfunction, anemia, and bone disease (CRAB).

In another seminal study by Walker et al., mutations in MM cases with t(4;14) and t(11;14) cytogenetics were compared to understand tumor evolution and treatment resistance [10]. Median acquired nonsynchronous exonic SNVs were found to be higher in cases with t(4;14) compared to t(11;14) (27 vs. 23.5), despite the absence of statistical significance, and the transition and transversion rates between both groups were comparable. Regardless of the driver status, RAS-MAPK pathway deregulation was common in both groups, and clones with this alteration were not always present in the dominant clone rather than in one or more subclonal populations. Furthermore, the persistent acquisition of mutations within subclones has led to the disease progression (Figure 3). 

## 8. Evolving Therapeutic Implications 

To date, MM is an incurable disease. Significant advancements have been made in translation and developmental therapeutic research to identify amenable genomic targets and develop novel agents that could potentially improve patients’ survival. However, these efforts have been severely challenged by the complex disease biology and molecular mechanisms of MM [99]. Detailed review on therapeutic implications in MM is beyond the scope of this article, but we will briefly describe some of the targeted agents that are currently explored [100]. BCL2 inhibitor Venetoclax as monotherapy and in combination with other anti-myeloma agents demonstrated improved outcome in early phases of clinical trials in patients with t(11;14) [101,102,103]. Molibresib (NCT01943851) and OTX015 (NCT01713582) are Bromodomain and Extra-Terminal motif (BET) inhibitors that have been actively investigated in patients with MYC translocations [104]. Poly (ADP-ribose) polymerase inhibitors (PARP) inhibitors such as Olaparib (NCT02693535, NCT03297606), veliparib (NCT01495351), and talazoparib (NCT02693535) targeting AMT and BRCA 1/2 mutations are in early phases of clinical trials. Vemurafenib (NCT03297606), encorafenib (NCT02834364), and dabrafenib (NCT03091257) are BRAF inhibitors which are also in early phase of drug development in combination with other agents. 

Fc receptor-homolog 5 (FcRH5), whose gene is located on chromosome 1, is a type I membrane protein expressed in MM cells. Cevostamab is a T-cell engaging bispecific antibody that targets FcRH5, and early pharmacodynamics studies described its mechanism of action which encompasses T-cell activation, proliferation and cytokine production [105]. A phase-1 clinical trial (NCT03275103) is currently ongoing to evaluate the safety and pharmacokinetics of Cevostamab (BFCR4350A) in relapsed/refractory MM. Initial results from the phase-1 clinical trial revealed the favorable activity of Cevostamab in heavily pre-treated relapsed/refractory MM [106] and whether 1q amp/gain MM may respond better to FcRH5 targeted by Cevostamab needs to be investigated further. In addition, immune effector therapies (at this early stage of clinical development) appear to be mutation agnostic; yet they may improve chances to control high-risk MM [107].

## 9. Conclusions

Multiple myeloma is a heterogeneous disease that is driven by numerous genetic and epigenetic changes. Early events in MM progression include immunoglobulin translocation and hyperdiploidy; however, the recent advancements in high throughput technologies (i.e., WGS) have identified complex genomic variations in key myeloma-associated genes responsible for the development and progression of the disease. In the future, data obtained from large cohorts of patients studied by newer genomic tools and analyzed with more sophisticated machine learning approaches will better characterize the genomic complexity and evolutionary changes responsible for disease progression. This will not only help us develop better prognostic models, but also identify novel therapeutic targets to develop individualized therapies. Indeed, prospective advances in the understanding of disease development from its precursor stage at the molecular level may help create novel avenues of rational preventive strategies that aim to avert early malignant clones from progression.

## Figures and Tables

**Table 1:** Revised International Staging System (R-ISS) stages according to Palumbo et al. [63].

**I**	Serum β2 microglobulin < 3.5 mg/L
	Serum albumin ≥ 3.5 g/dL
	Normal serum lactate dehydrogenase
	No high-risk cytogenetics *
**II**	Not meeting criteria for Stages I or III
**III**	Serum β2 microglobulin > 5.5 mg/L and at least one of the following:
	-Elevated serum lactate dehydrogenase
	-High-risk cytogenetics *

* High risk defined as: presence of del(17p) and/or translocation t(4;14) and/or translocation t(14;16) according to fluorescent in-situ hybridization (FISH) studies.
